# Meta-analysis of serum amyloid A for the diagnosis of neonatal sepsis: A comprehensive evaluation of diagnostic accuracy and clinical utility

**DOI:** 10.1097/MD.0000000000044197

**Published:** 2025-08-29

**Authors:** Haidao Wang, Jianfeng Yao, Baoshan Huang, Baiye Xu, Dengchao Wang

**Affiliations:** a Pediatrics, Quanzhou First Hospital Affiliated to Fujian Medical University, Quanzhou, Fujian Province, China; b Department of Gynaecology and Obstetrics, Quanzhou Maternity and Child Health Care Hospital, Quanzhou, Fujian Province, China; c Pediatrics, The Second Affiliated Hospital, Fujian Medical University, Quanzhou, Fujian Province, China; d Department of General Surgery, Zigong Fourth People’s Hospital, Zigong, Sichuan Province, China.

**Keywords:** meta-analysis, neonatal sepsis, serum amyloid A protein

## Abstract

**Background:**

Neonatal sepsis remains a major clinical challenge due to its high morbidity and mortality, necessitating reliable diagnostic markers for early detection. This meta-analysis evaluates the potential of serum amyloid A (SAA) as a diagnostic biomarker for neonatal sepsis, given its role in the acute inflammatory response.

**Methods:**

Computerized searches of PubMed, Embase, Cochrane Library, China National Knowledge Infrastructure, Wanfang Database, VIP Database, and China Biology Medicine disc (CBM) were conducted to collect relevant studies on the diagnostic role of SAA in neonatal sepsis. Literature screening, data extraction, and quality assessment of studies were performed according to inclusion and exclusion criteria and QUADAS standards. Data analysis was conducted using Stata 17.0 and Meta-Disc 1.4 software. Stata 17.0 was used to calculate the pooled sensitivity, specificity, positive likelihood ratio, negative likelihood ratio, and diagnostic odds ratio of included studies, and to draw forest plots and summary receiver operating characteristic (SROC) curves. The area under the SROC curve was calculated, and a funnel plot was constructed to assess publication bias.

**Results:**

A total of 16 articles, comprising 971 neonates with sepsis, were included. Meta-analysis results showed that the combined sensitivity and specificity of SAA in diagnosing neonatal sepsis were 0.85 (95% CI: 0.79–0.89) and 0.86 (95% CI: 0.77–0.92), respectively. The combined positive likelihood ratio and negative likelihood ratio were 6.3 (95% CI: 3.6–10.9) and 0.18 (95% CI: 0.12–0.25), respectively, and the diagnostic odds ratio was 36 (95% CI: 16–79). The area under the curve of the SROC curve was 0.91, and the Deek funnel plot suggested no publication bias in the included studies.

**Conclusion:**

SAA has high sensitivity and specificity in diagnosing neonatal sepsis, providing important evidence for its treatment. However, there is considerable heterogeneity among different studies, and further high-quality prospective studies are needed to confirm its clinical applicability.

## 1. Introduction

Neonatal sepsis is a common and challenging clinical condition, with high incidence and mortality rates globally. Due to the underdeveloped immune system and relatively weak resistance of neonates, infection can lead to severe complications and even life-threatening consequences.^[1,2]^ Its impact is not limited to immediate health issues but may also result in adverse neurodevelopmental outcomes and predispose infants to long-term diseases such as asthma and allergies.^[3–5]^ Timely identification of neonatal sepsis is crucial; however, traditional diagnostic methods like blood culture are time-consuming, and currently, there is no single ideal diagnostic biomarker.^[6,7]^ Differentiating novel or complementary biomarkers with early detection capability, high sensitivity, and specificity will aid in initiating appropriate treatment promptly while avoiding unnecessary interventions in noninfectious cases.

Early detection and treatment of sepsis are crucial for the survival and development of neonates in the field of neonatology. In recent years, SAA has garnered attention as a potential biomarker for the early diagnosis of neonatal sepsis. Previous studies have suggested that SAA levels may significantly increase during the acute inflammatory response, rendering it a potential diagnostic indicator.^[8–10]^ However, existing research results are inconsistent and controversial, with some studies having small sample sizes and insufficient evidence. Therefore, a comprehensive and objective assessment of the accuracy and clinical prospects of SAA in the diagnosis of neonatal sepsis is of great significance. In 2013, Yuan et al published a meta-analysis,^[11]^ which demonstrated the potential and meaningful value of SAA in the diagnosis of neonatal sepsis but only included 9 studies, with relevant research reports emerging in recent years. This study aims to comprehensively retrieve relevant studies in both Chinese and English languages and evaluate the value of SAA as a diagnostic indicator for neonatal sepsis through meta-analysis methods. We systematically screen relevant literature and pool data to obtain indicators such as the sensitivity and specificity of SAA in diagnosing neonatal sepsis. Through this research, we aim to provide clinicians with more reliable diagnostic evidence, thereby better guiding the early diagnosis and treatment of neonatal sepsis to improve neonate survival rates and quality of life.

## 2. Materials and methods

Our systematic review was conducted in accordance with the preferred reporting items for systematic reviews and meta-analyses for protocols (PRISMA-P)^[12]^ guidelines and is registered under the identifier INPLASY202470056.

### 2.1. Inclusion and exclusion criteria

Inclusion criteria: This study evaluates the diagnostic accuracy of SAA in neonatal sepsis, including both blood culture-positive sepsis and clinical sepsis cases. Sepsis is diagnosed if one or more clinical signs suggestive of infection are followed by positive blood, urine, or cerebrospinal fluid culture results, except for coagulase-negative staphylococci, which require 2 positive blood culture results. Clinical sepsis is diagnosed if one or more signs of infection and 2 laboratory indicators suggestive of sepsis persist for more than 24 hours but without positive culture results. Extraction of true positive (TP), true negative (TN), false positive (FP), and false negative (FN) values of SAA for diagnosing neonatal sepsis from literature retrieval.

Exclusion criteria: duplicate publications; publications lacking accessible original data needed to extract TP, TN, FP, and FN values; studies published in languages other than Chinese or English; research not involving neonates; study subjects are not neonates; animal studies.

### 2.2. Search strategy

We searched Chinese and English literature on the diagnosis of neonatal sepsis using SAA in PubMed, Embase, Cochrane Library, CNKI, Wanfang Database, VIP, and CBM from their inception to April 13, 2023. The search was conducted using a combination of MeSH terms and free-text terms, including “serum amyloid A protein,” “serum amyloid A,” “amyloid serum protein SAA,” “amyloid-related serum protein (SAA),” “serum A related protein,” “amyloid A precursor,” “amyloid protein SAA,” “amyloid A protein,” “amyloid fibril protein AA,” “amyloid protein AA,” “sepsis,” “bloodstream infection,” “pyemia,” “pyohemia,” “pyaemia,” “septicemia,” “blood poisoning,” “infant,” “newborn,” and “neonate.” Taking PubMed as an example, the search strategy was: ((((“serum amyloid A protein”[Mesh]) OR (((((((((serum amyloid A[Title/Abstract]) OR (amyloid serum protein SAA[Title/Abstract])) OR (amyloid-related serum protein (SAA[Title/Abstract]))) OR (serum A related protein[Title/Abstract])) OR (amyloid A precursor[Title/Abstract])) OR (amyloid protein SAA[Title/Abstract])) OR (amyloid A protein[Title/Abstract])) OR (amyloid fibril protein AA[Title/Abstract])) OR (amyloid protein AA[Title/Abstract]))) AND ((“infant, newborn”[Mesh]) OR ((newborn[Title/Abstract]) OR (neonate[Title/Abstract])))). Additionally, we reviewed the reference lists of included articles to identify further relevant studies meeting the inclusion criteria.

### 2.3. Literature screening and data extraction

Screening of literature was independently performed by 2 researchers using established inclusion and exclusion criteria, followed by cross-validation of the extracted data. Any disagreements were settled through discussion or with help from a third researcher. The data extracted comprised: general details such as the first author’s name, year of publication, country, sample sizes of the sepsis and control groups, age range, detection methods, and cutoff values; diagnostic outcomes including TP, FP, FN, and TN.

### 2.4. Quality assessment

Quality assessment of the studies incorporated into the meta-analysis was conducted using the QUADAS diagnostic test evaluation scale.^[13]^

### 2.5. Statistical analysis

Data analysis was performed with Stata 17.0 and Meta-Disc 1.4. Heterogeneity across the studies was examined using the Cochran-*Q* test and *I*^2^ statistic. A *P*-value <.05 and an *I*^2^ exceeding 50% necessitated the use of a random-effects model; otherwise, a fixed-effects model was applied.^[14]^ The analysis computed sensitivity, specificity, positive and negative likelihood ratios, and the diagnostic odds ratio for each study, each accompanied by 95% confidence intervals. Additionally, forest plots and summary receiver operating characteristic (SROC) curves were constructed, and the area under the curve (AUC) was estimated. Meta-Disc 1.4 facilitated the detection of threshold effects via Spearman correlation coefficient, indicative of significant correlation. Meta-regression and subgroup analyses were employed to further elucidate heterogeneity sources and variance in sensitivity and specificity across subgroups, respectively. The presence of publication bias was assessed using Deek funnel plot, with a significance threshold of *P* < .05.

### 2.6. Ethics

As a systematic review and meta-analysis based solely on previously published literature, with no involvement of new human experiments, patient recruitment, or collection of personally identifiable information, ethical approval was not required.

## 3. Results

### 3.1. Literature search results

**Figure 1.**
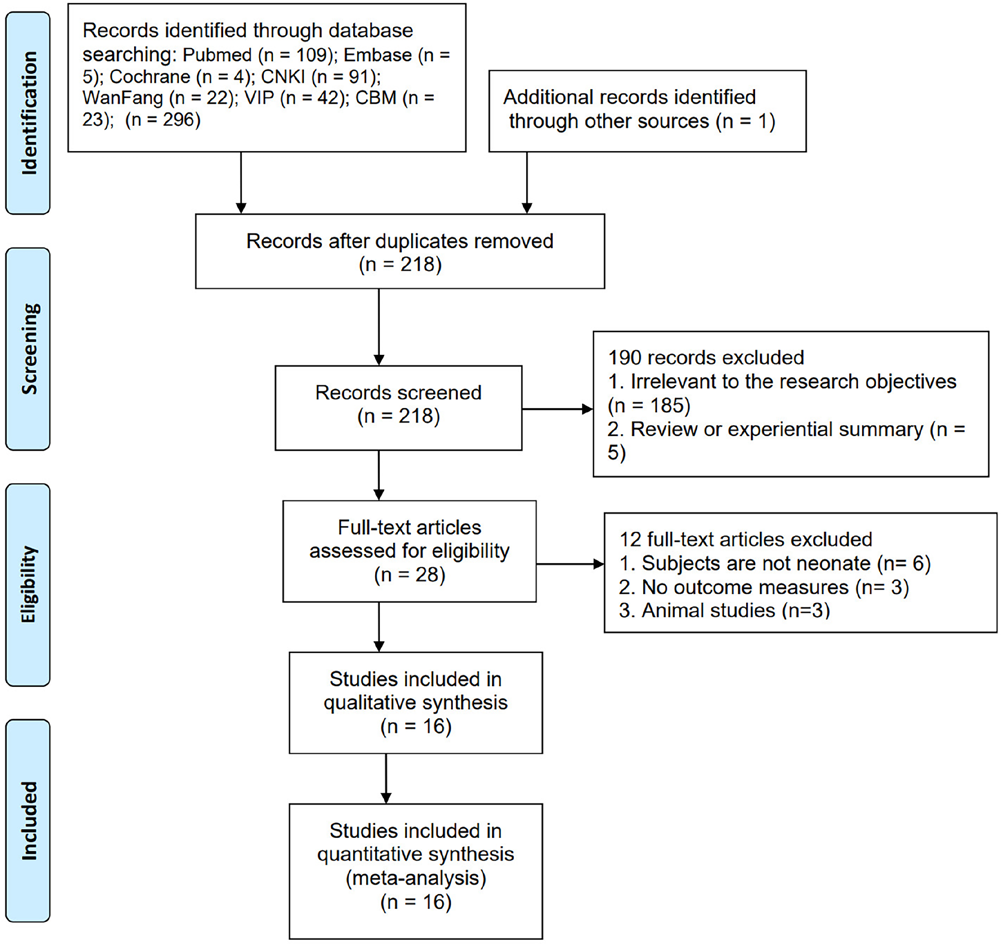
Study screening process.

Originally, a total of 296 articles were identified through database searches, and an additional 1 article was manually retrieved. After evaluating titles and abstracts, 79 duplicate articles and 185 articles unrelated to the research objectives were excluded, along with 5 reviews or experience summaries, 6 articles not involving neonates, 3 articles lacking outcome indicators, and 3 animal studies. Through stepwise screening, a total of 16 articles were finally included,^[15–30]^ involving 971 neonates with sepsis. Figure 1 illustrates the detailed screening process, and Table 1 displays the basic characteristics of the studies included.

**Table 1:** Basic characteristics of included studies.

Study	Country	Sample size (M/F)		Age (wk)		Detection methods	Cutoff (mg/L)	TP	FP	FN	TN
		Septic group	Control group	Septic group	Control group						
Abd Elkhalek 2020^[15]^	Egypt	45 (30/15)	40 (20/20)	35.4 ± 2.3	36.7 ± 1.9	ELISA	2.8	39	6	6	34
Arnon 2002^[16]^	Israel	42 (Na/Na)	37 (Na/Na)	Na	Na	ELISA	10	42	2	0	35
Arnon 2004^[17]^	Israel	38 (19/19)	78 (40/38)	10 (5–36)	14 (4–32)	ELISA	10	36	5	2	73
Arnon 2007^[18]^	Israel	23 (13/10)	81 (37/44)	39.5 ± 1.1	38.2 ± 1.1	Automated latex photometric immunoassay agglutination reaction	8	22	4	1	77
Bourika 2020^[19]^	Greece	113 (80/33)	68 (46/22)	38.8 ± 1.4	38.4 ± 1.7	Immunonephelometry using the BN ProSpec system	11.3	88	0	25	68
Çetinkaya 2009^[20]^	Turkey	123 (68/55)	40 (22/18)	31.2 ± 3.2	32.7 ± 1.6	Immunonephelometric method with the BN II device	68	94	0	29	40
Edgar 2010^[21]^	Northern Ireland	36 (Na/Na)	27 (Na/Na)	24–41	24–41	ELISA	1	23	10	13	17
Enguix 2011^[22]^	Spain	20 (Na/Na)	26 (Na/Na)	Na	Na	Automatic laser nephelometry	41.3	20	6	0	24
Fu 2022^[23]^	China	113 (67/56)	100 (68/32)	34.1 ± 1.1	34.3 ± 1.1	ELISA	34.8	97	8	16	92
Pan 2022^[24]^	China	32 (16/16)	32 (18/14)	0.3–3*	0.3–3*	Nephelometric assay	10	24	5	8	27
Sharma 2023^[25]^	India	28 (Na/Na)	46 (Na/Na)	32.8 ± 3.7	32.8 ± 3.7	Enhanced immune turbidimetric assay and immunochromatography	10.3	23	18	5	28
Ucar 2008^[26]^	Turkey	36 (23/13)	36 (24/12)	34.5 ± 0.5	33.4 ± 0.4	ELISA	6.6	27	20	9	16
Wang 2013^[27]^	China	35 (Na/Na)	45 (Na/Na)	39.0 ± 1.2	38.9 ± 0.8	ELISA	5.3	30	9	5	36
Wang 2016^[28]^	China	40 (23/17)	40 (25/15)	39.3 ± 1.1	39.8 ± 1.0	ELISA	5.3	34	8	6	32
Wu 2019^[29]^	China	195 (107/88)	100 (53/47)	28.8 ± 9.2	29.4 ± 9.7	ELISA	69.28	162	34	33	66
Zhao 2018^[30]^	China	52 (30/22)	30 (19/11)	38.9 ± 1.2	38.5 ± 1.2	ELISA	49.52	42	5	10	25

Na = not available, FN = false negatives, FP = false positives, TN = true negatives, TP = true positives.

* Age in days.

### 3.2. Quality assessment results of included studies

Table 2 presents the results of the quality assessment for the included studies.

**Table 2:** Quality assessment results of included studies.

Study	Q1	Q2	Q3	Q4	Q5	Q6	Q7	Q8	Q9	Q10	Q11	Q12	Q13	Q14
Abd Elkhalek 2020^[15]^	√	√	√	√	√	√	√	√	√	?	?	√	×	×
Arnon 2002^[16]^	√	×	√	?	√	√	√	×	×	?	?	√	×	×
Arnon 2004^[17]^	√	×	√	?	√	√	√	×	×	?	?	√	×	×
Arnon 2007^[18]^	√	√	√	?	√	√	√	√	×	√	√	√	√	×
Bourika 2020^[19]^	√	×	√	√	√	√	√	×	√	?	?	√	×	×
Çetinkaya 2009^[20]^	√	√	√	√	√	√	√	×	√	?	?	√	×	×
Edgar 2010^[21]^	√	√	√	√	√	√	√	×	√	√	√	√	√	×
Enguix 2011^[22]^	√	×	√	?	√	√	√	×	×	?	?	√	×	×
Fu 2022^[23]^	√	√	√	√	√	√	√	√	√	?	?	√	×	×
Pan 2022^[24]^	√	√	√	?	√	√	√	×	×	?	?	√	×	×
Sharma 2023^[25]^	√	√	√	√	√	√	√	√	√	?	?	√	×	×
Ucar 2008^[26]^	√	×	√	?	√	√	√	√	×	?	?	√	×	×
Wang 2013^[27]^	√	×	√	√	√	√	√	√	√	?	?	√	×	×
Wang 2016^[28]^	√	√	√	√	√	√	√	√	√	?	?	√	×	×
Wu 2019^[29]^	√	√	√	?	√	√	√	√	×	?	?	√	×	×
Zhao 2018^[30]^	√	√	√	?	√	√	√	√	×	?	?	√	×	×

√: yes; ×: no;?: uncertain;

Evaluation criteria:

1: Does the case spectrum include various cases and cases of confusing diseases?

2: Is the selection of study subjects accurately and clearly defined in terms of inclusion and exclusion criteria?

3: Can the gold standard accurately distinguish between diseased and non-diseased states?

4: Is the interval between the gold standard and the evaluated test short enough to avoid changes in the disease condition?

5: Did all samples or randomly selected samples undergo the gold standard test?

6: Did all cases undergo the same gold standard test regardless of the results of the evaluated test?

7: Is the gold standard test independent of the evaluated test (i.e., the evaluated test is not included in the gold standard)?

8: Is the operation of the evaluated test described clearly enough and replicable?

9: Is the operation of the gold standard test described clearly enough and replicable?

10: Was the interpretation of the results of the evaluated test performed without knowing the results of the gold standard test?

11: Was the interpretation of the results of the gold standard test performed without knowing the results of the evaluated test?

12: Are the clinical data available for interpretation of the test results consistent with the clinical data available in actual practice?

13: Are difficult to interpret/ intermediate test results reported?

14: Are explanations provided for cases that dropped out of the study?

### 3.3. Heterogeneity and threshold effect analysis

For the diagnosis of neonatal sepsis using SAA, the I^2^ values for sensitivity and specificity were 67.05% and 88.80%, respectively. Cochran-*Q* test yielded *P*-values of .00 for both, indicating significant heterogeneity among the studies, thus necessitating a threshold effect analysis. Utilizing Meta-Disc 1.4 software, the Spearman correlation coefficient was determined to be −0.05 with a *P*-value of .86, indicating no threshold effect heterogeneity. Based on these findings, a random-effects model was employed to estimate the combined effect sizes.

### 3.4. Meta-analysis results

**Figure 2.**
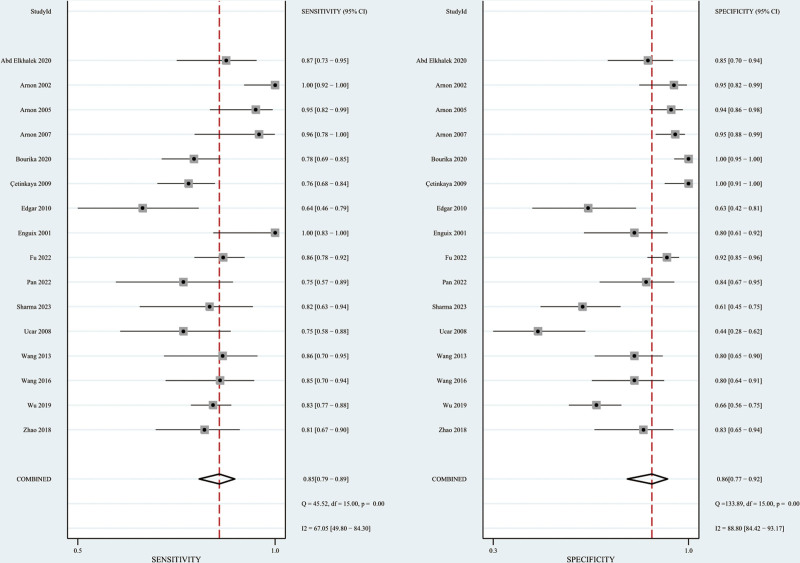
Forest plot of sensitivity and specificity for SAA diagnosis of neonatal sepsis. SAA = serum amyloid A

**Figure 3.**
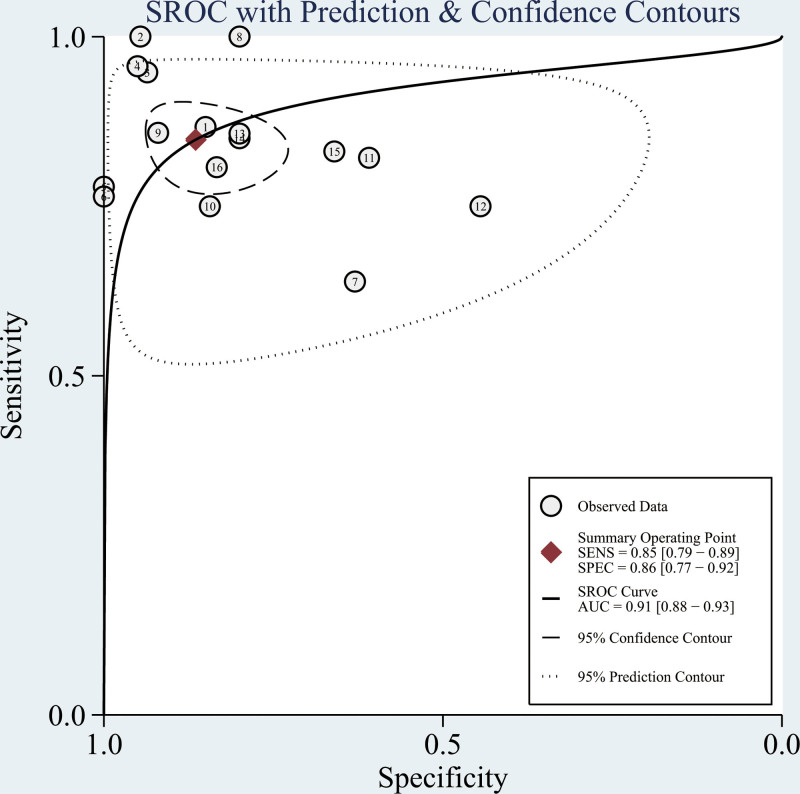
Receiver operating characteristic curve for SAA diagnosis of neonatal sepsis. SAA = serum amyloid A.

Using Stata 17.0, Forest plots and SROC curves were created to display the diagnostic accuracy of SAA for neonatal sepsis. Calculations included combined sensitivity and specificity, positive and negative likelihood ratios, diagnostic odds ratio, and the AUC. Specifically, sensitivity was 0.85 (95% CI: 0.79–0.89) and specificity was 0.86 (95% CI: 0.77–0.92). The positive and negative likelihood ratios were 6.3 (95% CI: 3.6–10.9) and 0.18 (95% CI: 0.12–0.25), respectively. The diagnostic odds ratio reached 36 (95% CI: 16–79), and the AUC for the SROC curve stood at 0.91. Figures 2 and 3 illustrate the forest plot and SROC curve, respectively, for assessing neonatal sepsis through SAA.

### 3.5. Publication bias

**Figure 4.**
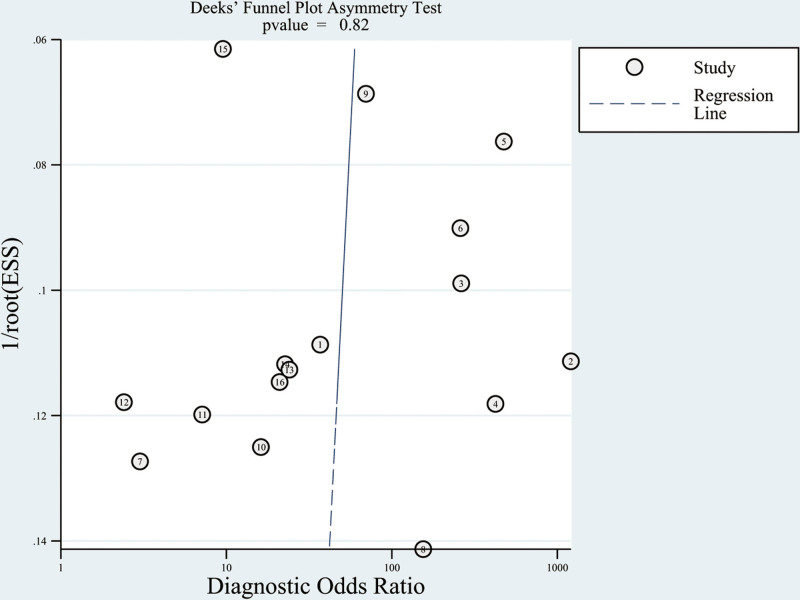
Deek funnel plot.

A Deek funnel plot was constructed to evaluate publication bias in studies assessing neonatal sepsis diagnosis via SAA, demonstrating general symmetry. The linear regression test for funnel plot asymmetry returned a *P* value of .82, indicating a low likelihood of publication bias (Fig. 4).

### 3.6. Meta-regression and subgroup analysis

Heterogeneity due to threshold effects has been excluded. Therefore, single-factor meta-regression analysis was conducted using country, research time, sample size of sepsis, cutoff value of SAA, detection method, and blinding as factors. The results showed that country, research time, sample size of sepsis, cutoff value of serum amyloid A, and detection method were the main factors contributing to the heterogeneity in sensitivity, while no main factors contributing to heterogeneity in specificity were found. Subgroup analysis results are presented in Table 3.

**Table 3:** Subgroup analysis results of included studies.

Factor	Subgroup	Number of studies	Sensitivity (95% CI)	*P*-value	Specificity (95% CI)	*P*-value
Country	Non-China	10	0.86 (0.80–0.92)	.02	0.89 (0.80–0.97)	.69
	China	6	0.83 (0.76–0.91)		0.83 (0.69–0.96)	
Research time	Within the past 10 yr	8	0.87 (0.81–0.94)	.04	0.87 (0.78–0.97)	.37
	More than 10 yr ago	8	0.83 (0.76–0.89)		0.85 (0.75–0.96)	
Sample size of sepsis	≤100	5	0.81 (0.73–0.90)	.00	0.93 (0.85–1.00)	.84
	>100	11	0.87 (0.82–0.93)		0.82 (0.72–0.92)	
cutoff value of serum	≤10 mg/L	7	0.83 (0.76–0.90)	.00	0.89 (0.79–0.98)	.55
Amyloid A	>10 mg/L	9	0.86 (0.80–0.92)		0.84 (0.74–0.95)	
Detection method	Non-ELISA	6	0.83 (0.74–0.91)	.00	0.92 (0.84–0.99)	.98
	ELISA	10	0.85 (0.80–0.91)		0.82 (0.72–0.93)	
Blinding was used in the research process	No	14	0.85 (0.80–0.90)	.43	0.87 (0.79–0.94)	.98
	Yes	2	0.81 (0.64–0.97)		0.85 (0.64–1.00)	

CI = confidence interval.

## 4. Discussion

Neonatal sepsis is a disease that continues to pose a challenge to us, characterized by diverse symptoms, difficult prediction, and challenging diagnosis. Without proper treatment, it can rapidly progress and lead to death or disability, making it a major health concern worldwide.^[31,32]^ Despite this, the diagnosis of neonatal sepsis remains challenging, with varied clinical presentations, uncertain laboratory tests, and complex treatment processes.^[33,34]^ Currently used biomarkers such as IL-6, IL-8, PCT, and CRP have limitations in diagnosing neonatal sepsis, lacking an ideal marker to accurately differentiate sepsis from other conditions.^[35]^ While some new biomarkers like SAA have been proposed, their clinical application still presents challenges, requiring further research to validate their accuracy and reliability. Therefore, finding an accurate and reliable diagnostic method has become an urgent need in the medical field.

SAA, as an inflammation marker, significantly increases during the process of inflammation and has been widely investigated for the diagnosis of infectious diseases.^[36]^ This study aims to systematically evaluate the value of SAA in the diagnosis of neonatal sepsis using meta-analysis. Sixteen articles, covering a total of 971 neonatal sepsis patients, were included for a comprehensive assessment of SAA’s performance in the diagnosis of neonatal sepsis. The results showed that the sensitivity and specificity were 0.85 and 0.86, respectively, indicating a relatively high accuracy of SAA in the diagnosis of neonatal sepsis. This may be attributed to the rapid release of SAA as an early inflammation marker in sepsis, with its concentration changes able to accurately reflect the presence of infection in patients. The pooled positive likelihood ratio was 6.3, and the pooled negative likelihood ratio was 0.18. A higher positive likelihood ratio implies a greater likelihood of sepsis when a positive result occurs, while a lower negative likelihood ratio suggests a smaller likelihood of sepsis when a negative result occurs. The diagnostic odds ratio was 36, indicating the odds of neonates having sepsis compared to not having sepsis. A higher diagnostic odds ratio demonstrates the superiority of SAA as a diagnostic marker in distinguishing sepsis from non-sepsis. The area under the SROC curve (AUC) was 0.91, indicating a relatively high overall diagnostic accuracy of SAA in the diagnosis of neonatal sepsis. The high AUC value of SAA indicates its good discriminative ability as a biomarker in diagnosis, which contributes to improving diagnostic accuracy and reliability. SAA is an acute-phase protein that is typically significantly upregulated during inflammation and infection states. Its diagnostic value in neonatal sepsis may be associated with several biological mechanisms as follows: Increased expression of SAA due to enhanced inflammatory response: Neonatal sepsis results from severe inflammatory responses caused by infection, leading to increased production of SAA. The upregulation of SAA under this inflammatory condition may serve as the basis for its role as a biomarker.^[37,38]^ Association of SAA with immune regulation: SAA not only participates in the inflammatory response but also may influence immune regulation processes. In sepsis, immune function is compromised, and SAA may be involved in disease occurrence and progression by modulating immune cell function or mediating immune responses.^[39,40]^ Relationship between SAA and bacterial clearance and immune response: Some studies suggest that SAA may participate in the clearance of bacteria or regulate some key steps in the immune response. Therefore, the levels of SAA may reflect the body’s response status to bacterial infection.^[41,42]^ Interaction of SAA with inflammatory mediators: SAA may interact with other inflammatory mediators such as tumor necrosis factor-alpha (TNF-α), interleukins (ILs), etc.^[39,43]^ These interactions may further affect the expression levels and diagnostic value of SAA in neonatal sepsis.

This study also has certain limitations: there is a high heterogeneity among different studies, which may affect the stability and consistency of the results; some literature did not describe whether blinding was used for interpreting the gold standard, which may introduce bias in result interpretation; only Chinese and English literature were included, which may lead to publication bias to some extent. The majority of the literature authors and cases are from China, suggesting a potential bias; different studies have inconsistent detection methods and cutoff values; all these factors may decrease the reliability of the study results, and future large-scale standardized prospective studies are still needed to confirm the value of SAA in the diagnosis of neonatal sepsis.

## 5. Conclusions

In conclusion, SAA demonstrates high diagnostic efficacy for the diagnosis of neonatal sepsis, providing clinicians with an effective diagnostic tool that aids in the early detection of sepsis, formulation of appropriate treatment plans, and consequently, improvement of treatment outcomes and survival rates associated with sepsis.

## Author contributions

**Conceptualization:** Haidao Wang, Jianfeng Yao, Baoshan Huang, Dengchao Wang.

**Data curation:** Haidao Wang, Jianfeng Yao, Baoshan Huang, Baiye Xu.

**Formal analysis:** Haidao Wang, Baoshan Huang, Baiye Xu.

**Funding acquisition:** Haidao Wang, Baoshan Huang.

**Investigation:** Haidao Wang, Baoshan Huang.

**Methodology:** Haidao Wang, Baoshan Huang, Baiye Xu, Dengchao Wang.

**Project administration:** Haidao Wang, Baoshan Huang, Dengchao Wang.

**Resources:** Haidao Wang, Jianfeng Yao, Baoshan Huang, Baiye Xu, Dengchao Wang.

**Software:** Haidao Wang, Baoshan Huang.

**Supervision:** Baoshan Huang, Dengchao Wang.

**Validation:** Haidao Wang, Jianfeng Yao, Baoshan Huang, Baiye Xu, Dengchao Wang.

**Visualization:** Haidao Wang.

**Writing – original draft:** Haidao Wang, Jianfeng Yao, Baoshan Huang, Baiye Xu, Dengchao Wang.

**Writing – review & editing:** Haidao Wang, Jianfeng Yao, Baoshan Huang, Baiye Xu, Dengchao Wang.
